# Degradation of clofibric acid in UV/chlorine disinfection process: kinetics, reactive species contribution and pathways

**DOI:** 10.1098/rsos.171372

**Published:** 2018-02-07

**Authors:** Yuqing Tang, Xueting Shi, Yongze Liu, Li Feng, Liqiu Zhang

**Affiliations:** Beijing Key Lab for Source Control Technology of Water Pollution, College of Environmental Science and Engineering, Beijing Forestry University, Beijing 100083, People's Republic of China

**Keywords:** UV/chlorine disinfection process, clofibric acid, reactive chlorine species, degradation kinetics and pathway

## Abstract

As a potential endocrine disruptor, clofibric acid (CA) was investigated in this study for its degradation kinetics and pathways in UV/chlorine process. The results showed that CA in both UV photolysis and UV/chlorine processes could be degraded via pseudo-first-order kinetics, while it almost could not be degraded in the dark chlorination process. The observed rate constant (*k*_obs_) in UV photolysis was 0.0078 min^−1,^ and increased to 0.0107 min^−1^ combining with 0.1 mM chlorine. The *k*_obs_ increased to 0.0447 min^−1^ with further increasing the chlorine dosage from 0.1 to 1.0 mM, and reached a plateau at higher dosage (greater than 1.0 mM). The higher *k*_obs_ was obtained at acid solution rather than basic solution. Moreover, the calculated contributions of radical species to *k*_obs_ indicated that the HO• contributed significantly to CA degradation in acidic conditions, while the reactive chlorine species and UV direct photolysis dominated in neutral and basic solution. The degradation of CA was slightly inhibited in the presence of $\text{HC}{{\text{O}}_{3}}^{-}$ (1 ∼ 50 mM), barely affected by the presence of Cl^−^ (1 ∼ 200 mM) and greatly suppressed by humic acid (0 ∼ 5 mg l^−1^). Thirteen main degradation intermediates and three degradation pathways of CA were identified during UV/chlorine process.

## 1. Introduction

Pharmaceutical and personal care products (PPCPs) are becoming ubiquitous in the environment and have been frequently detected in wastewater, seawater, surface water and even drinking water around the world [1]. Many of them have potential impacts on aquatic organisms and their long-term existence may cause a threat to human health [2]. As a representative cholesterol-lowering drug, clofibric acid (CA) is considered as a potential endocrine disruptor because it interferes with the synthesis of cholesterol [3]. Owing to the complex structures of CA and its intermediates, biological degradation in the environment is insufficient to remove these chemicals. CA is one of the most persistent PPCPs known, with an estimated environmental residence of 21 years [4]. Therefore, CA has a high environmental persistence and becomes a most commonly detected drug at concentrations of 270–660 ng l^−1^ in the water environment [5–7].

Current traditional wastewater and drinking water treatment processes, such as coagulation, sedimentation and filtration, and biological processes, do not efficiently remove CA [8]. It was reported that ultraviolet (UV) photolysis could efficiently degrade CA in wastewater [9]. Generally, UV photolysis is used as the disinfection process in wastewater treatment plant [10]. However, UV photolysis is an instantaneous disinfection process, without the residual disinfection effect of chlorination [11,12]. Therefore, UV photolysis is always combined with chlorine to achieve an efficient and continuous disinfection effect [13], i.e. UV/chlorine process. In recent several years, UV/chlorine process has attracted great interest due to its potential degradation of PPCPs [14,15] along with the disinfection effect. It has been reported that UV/chlorine process is effective in degrading many contaminants including trichloroethylene [16], desethylatrazine, sulfamethoxazole, carbamazepine [15] and diclofenac [17]. There is great concern about CA degradation in UV/chlorine process, which has not been studied so far.

Additionally, during UV/chlorine process, chlorine is activated by UV photolysis to produce highly reactive species (such as hydroxyl radical (HO•) and reactive chlorine species (RCS, such as $\text{Cl}∙/\text{C}{{\text{l}}_{2}}^{∙-}$)) (reaction (1.1–1.2)) [18,19], which might be responsible for the degradation of those PPCPs. These reactive species react with PPCPs via different pathways yielding distinct degradation products. It was quite important to investigate the relative contributions of these highly reactive species to PPCPs degradation.

1.1 $$\text{HOCl/OC}{\text{l}}^{-}+hv\to \text{HO}∙/{\text{O}}^{∙-}+\text{Cl}∙$$

and

1.2 $$\text{Cl}∙+{\text{Cl}}_{2}^{∙-}⇌\text{C}{{\text{l}}_{2}}^{-},\;k_{+}=\text{6.5}\times \text{1}{\text{0}}^{9}\;{\text{M}}^{-1}{\text{s}}^{-1}\text{, }\;k_{-}=\text{1.1}\times \text{1}{\text{0}}^{5}\;{\text{s}}^{-1}.$$

This paper firstly investigated the degradation kinetics of CA in UV/chlorine process. The effects of water matrix (such as chlorine dosage, pH, bicarbonates ($\text{HC}{{\text{O}}_{3}}^{-}$), chloride ion (Cl^−^) and humic acid (HA)) on CA degradation in UV/chlorine process were also evaluated. Furthermore, the important contributions of HO•, RCS and UV photolysis to CA degradation were assessed. Finally, the degradation products were monitored and the degradation pathways of CA in UV/chlorine process were proposed.

## 2. Material and methods

### 2.1. Chemicals and materials

CA (purity greater than 98%) was obtained from J&K Scientific, Ltd (Beijing, China). The CA stock solution (500 µM) was prepared by dissolving the pure compound in ultrapure water and stored at 4°C protected from light. Sodium hypochlorite solution (NaOCl) with 8% available chlorine was obtained from Xilong Chemical Co., Ltd (Beijing, China). Chromatographic grade chemicals of nitrobenzene (NB, purity > 99%), methanol and acetonitrile were purchased from Beijing Chemical Factory. All other reagents (NaOH, HClO_4_, KH_2_PO_4_, NaCl, *tert*-butanol (*t*BuOH), NaHCO_3_, KH_2_PO_4_, etc.) were analytical grade or above and were used without further purification. All solutions were prepared with ultrapure water (18.2 MΩ cm) produced from Water Purification System (Elga Purelab Classic, Veolia).

### 2.2. UV irradiation

**Figure 1.**
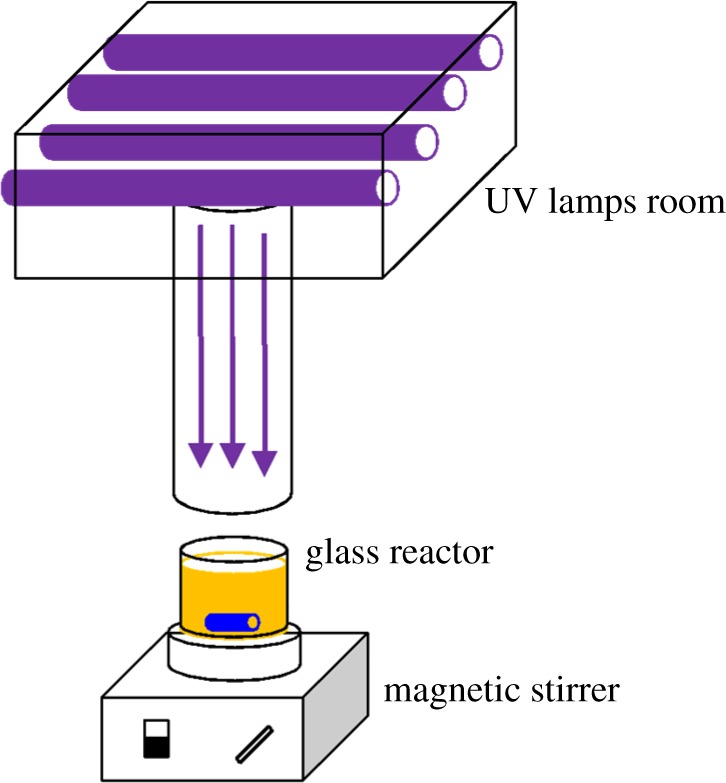
Collimated beam apparatus for UV photolysis.

Figure 1 shows a quasi-collimated beam apparatus used in this study. Four low-pressure (LP) UV lamps (Heraeus, GPH 212T5 L/4, 10 W, 254 nm) were placed 30 cm above the 100 ml photoreactor with rapid mixing at 400 r.p.m. with magnitude stirrer. The volume of the glass reactor is 100 ml, the inner diameter is 5.65 cm and the solution depth is 4.0 cm. The average surface irradiance was measured to be 0.16 mW cm^−2^ by a UV-B Ultraviolet radiometer (Beijing Normal University photoelectric instrument factory, Beijing, China) [20].

### 2.3. Experimental procedures

All experiments were carried out in the 100 ml photoreactor described above. The degradation of CA by UV/chlorine was initiated by spiking the specific amount NaOCl stock solution into CA solution (10 µM) to form the testing solution, and placing the testing solution under the UV irradiance beam. Control tests of CA degradation by UV photolysis and dark chlorination were conducted in similar procedure without UV irradiance or NaOCl solution, respectively. The reaction solution pH was buffered by 0.5 mM KH_2_PO_4_, and the initial pH was adjusted to specific value using 1 mM NaOH and 1 mM HClO_4_. Specific amounts of Cl^−^ (0 ∼ 200 mM), $\text{HC}{{\text{O}}_{3}}^{-}$ (0 ∼ 50 mM) and HA (0 ∼ 5 mg l^−1^) were added to the testing solution to examine their effects on CA degradation. To calculate the contributions of reactive radicals to CA degradation, 10 µM NB was added into the testing solution as a HO• probe. At selected time intervals, 1.5 ml sample was collected from the photoreactor and immediately quenched by addition of 0.1 mM ascorbic acid and stored at 4°C in the dark before analysis. All the experiments were performed in duplicate and relative standard deviations of all data points were below 5%. In order to better analyse CA degradation products in UV/chlorine process, higher CA concentration (0.3 mM) and chlorine dosage (1.6 mM) were applied to enhance the formation of degradation intermediates.

### 2.4. Analytical methods

The concentrations of free chlorine were determined using the DPD (*N*,*N*-diethyl-*p*-phenylenediamine, Sigma Aldrich, greater than 99%) colorimetric method [21]. The concentrations of CA and NB were analysed by a rapid resolution liquid chromatography system (RRLC, Agilent 1260 Infinity) equipped with a variable wavelength UV detector. A sample of 10 µl volume was injected onto a Poroshell 120 EC-C_18_ column (4.6 × 50 mm, 2.7 mm, Agilent, China). The CA and NB were detected at 227 nm and 262 nm, respectively. The flow rate of the mobile phase was 1 ml min^−1^, consisting of methanol/0.01% acetic acid/acetonitrile (v/v/v = 5 : 30 : 65) for CA determination and water/methanol (v/v = 50 : 50) for NB determination. The temperature of the column was maintained at 30°C. The degradation products of CA during UV/chlorine process were identified by high performance liquid chromatography (Ultimate 3000, Thermo Scientific, USA) with QE-MS/MS (QExactive plus, Thermo Scientific, USA). The separation column was a C_18_ column (2.1 mm × 100 mm, 1.7 µm, Waters, China). The mobile phase flow rate was 0.2 ml min^−2^ and the elution gradient was as follows: 0–10.0 min, 10% A (acetonitrile) and 90% B (pure water); 10.0–15.0 min, A increased from 10 to 50%; 15.0–22.0 min, A continued to increase from 50 to 99%; 22.0–25.0 min, A maintained at 99%; 25.0–30.0 min, A decreased to 10%.

## 3. Results and discussion

### 3.1. Degradation of CA in UV/chlorine process

**Figure 2.**
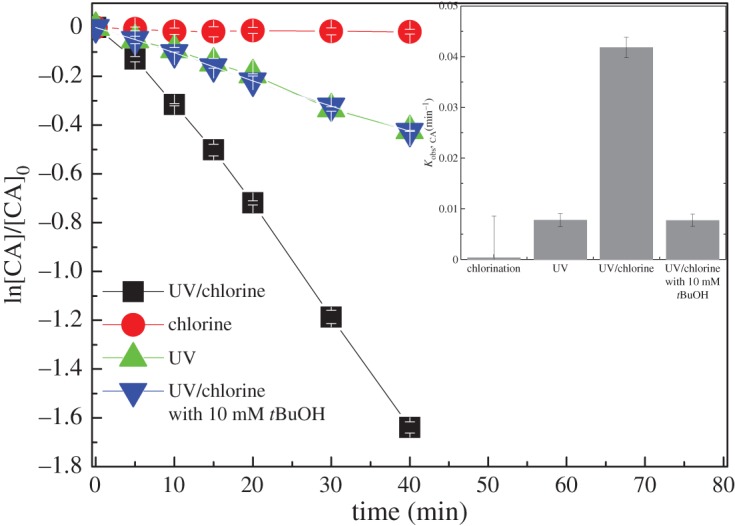
CA degradation in UV photolysis, dark chlorination and UV/chlorine processes. Experimental conditions: *I*_0_ = 0.16 mW cm^−2^, [Chlorine]_0_ = 0.8 mM, [CA]_0_ = 10 µM, pH = 7.0, *T* = 20°.

The time-dependent degradations of CA in UV photolysis, dark chlorination and UV/chlorine processes were compared. As shown in figure 2, dark chlorination could hardly degrade CA at 0.8 mM dosage, but both UV photolysis and UV/chlorine processes could degrade CA efficiently. The CA degradation in UV photolysis and UV/chlorine processes could be well fitted by pseudo-first-order kinetics. The observed rate constants (*k*_obs,CA_) of CA by UV photolysis was obtained to be 0.0078 min^−1^(table 1), and greatly increased to 0.0418 min^−1^ (table 1) by the combination of UV with 0.80 mM chlorine.

**Table 1.** The observed rate constant (*k*_obs_) for CA degradation under various experimental conditions.

figure no.	CA_0_ (μM)	[chlorine]_0_ (mM)	*I*_0_ mW cm^−2^	pH_0_	*k* (min^−1^)	*t*_1/2_ (min)	s.e.
2: Effect of disinfection process							
dark chlorination	10	0.8		7	0.0004	1732.2	0.0001
UV photolysis	10		0.16	7	0.0078	88.9	0.00028
UV/chlorine	10	0.8	0.16	7	0.04182	16.6	0.00145
UV/chlorine with 10 mM *t*BuOH	10	0.8	0.16	7	0.00778	89.1	0.00031
3: Effect of chlorine dosages							
	10	0.1	0.16	7	0.0107	64.8	0.0013
	10	0.3	0.16	7	0.0206	33.7	0.0009
	10	0.5	0.16	7	0.0373	18.6	0.0013
	10	0.8	0.16	7	0.0418	16.6	0.0026
	10	1.5	0.16	7	0.0447	15.6	0.0015
	10	2.0	0.16	7	0.0458	15.2	0.0027
4: Effect of initial pH							
	10	0.8	0.16	5	0.07951	8.7	0.01118
	10	0.8	0.16	6	0.0531	13.0	0.00436
	10	0.8	0.16	7	0.0318	21.8	0.00359
	10	0.8	0.16	8	0.02058	33.7	0.00423
	10	0.8	0.16	9	0.01702	40.7	0.00272
	10	0.8	0.16	10	0.0168	41.3	0.00323
5: Effect of HO• scavenger (NB)							
[NB] = 10 µM	10	0.8	0.16	5	0.06616		0.0035
[NB] = 10 µM	10	0.8	0.16	6	0.02979		0.0005
[NB] = 10 µM	10	0.8	0.16	7	0.00438		0.0004
[NB] = 10 µM	10	0.8	0.16	8	0.00429		0.0004
[NB] = 10 µM	10	0.8	0.16	9	0.00125		0.0001
[NB] = 10 µM	10	0.8	0.16	10	0.00082		0.0001
6: Effect of chloride							
[NaCl] = 50 mM	10	0.8	0.16	7	0.0379	18.3	0.00129
[NaCl] = 100 mM	10	0.8	0.16	7	0.03974	17.4	0.00118
[NaCl] = 200 mM	10	0.8	0.16	7	0.03929	17.6	0.00109
7: Effect of bicarbonate							
[NaHCO_3_] = 10 mM	10	0.8	0.16	7	0.03739	18.5	0.00112
[NaHCO3] = 20 mM	10	0.8	0.16	7	0.03534	19.6	0.00101
[NaHCO3] = 50 mM	10	0.8	0.16	7	0.03269	21.2	0.00132
8: Effect of HA							
[HA] = 1 mg l^−1^	10	0.8	0.16	7	0.0347	20.0	0.00092
[HA] = 5 mg l^−1^	10	0.8	0.16	7	0.00699	99.2	0.0003

Free chlorine absorbs photons to produce reactive radicals including HO• and RCS (reaction (1.1–1.2)). Thus, compared with UV photolysis, the greatly enhanced degradation of CA in UV/chlorine process could primarily be attributed to the formation of reactive radicals. With the addition of 10 mM *t*BuOH in UV/chlorine process, the CA degradation was greatly inhibited and comparable with that in UV photolysis. The *t*BuOH is a typical radical scavenger for HO• and Cl• with second-order rate constants of 6 × 10^8^ and 1.9 × 10^9^ M^−1^ s^−1^, respectively [15]. The rate constants of CA degradation by the UV direct photolysis and reactive radicals were calculated to be 0.0078 and 0.034 min^−1^, respectively. Thus, in UV/chlorine process at 0.8 mM chlorine dosage, the contributions of UV direct photolysis and reactive radicals to CA degradation could be determined to be 18.7% and 81.3%, respectively.

### 3.2. Effects of chlorine dosage on CA degradation in UV/chlorine process

**Figure 3.**
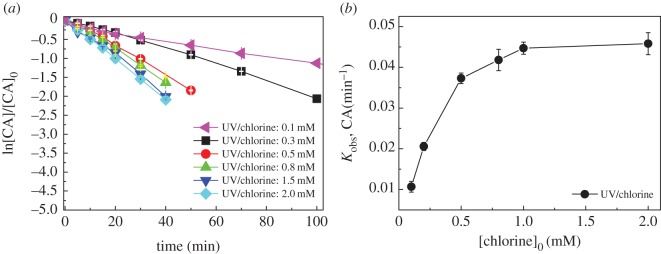
The degradation kinetics (*a*) and *k*_obs,CA_ (*b*) of CA at different chlorine dosages in UV/chlorine process. Experimental conditions: *I*_0_ = 0.16 mW cm^−2^, [CA]_0_ = 10 µM, pH = 7.0, *T* = 20°C.

The effects of chlorine dosage (0.1 ∼ 2.0 mM) on CA degradation in UV/chlorine process are shown in figure 3*a*. The degradation of CA at all chlorine dosages could be well fitted by pseudo-first-order kinetics and *k*_obs,CA_ was obtained, as shown in figure 3*b*. The *k*_obs,CA_ rapidly increased from 0.0107 to 0.037 min^−1^ (table 1) with the increase of chlorine dosage from 0.1 to 0.5 mM, and gradually increased to 0.0447 min^−1^ with further increasing the chlorine dosage from 0.5 to 1.0 mM, then reached plateaus (0.0458 min^−1^, table 1) at higher chlorine dosage (2.0 mM). The enhanced effect of chlorine dosage on CA degradation could be attributed to two reasons. On the one hand, photolysis of chlorine generates reactive radicals (reaction (1.1–1.2)) to accelerate CA degradation. On the other hand, chlorine can react with HO• and Cl• (reaction (3.1–3.4)), acting as scavenger of these reactive radicals [19,22,23].

3.1 $$\begin{array}{r} \text{HO}∙+\text{HOCl}\to \text{ClO}∙+{\text{H}}_{2}\text{O, }\;k_{3}=\text{2.0}\times \text{1}{\text{0}}^{9}\;{\text{M}}^{-1}\;{\text{s}}^{-1}, \end{array}$$

3.2 $$\begin{array}{r} \text{HO}∙+\text{OC}{\text{l}}^{-}\to \text{ClO}∙+\text{O}{\text{H}}^{-}\text{, }\;k_{4}=\text{8.8}\times \text{1}{\text{0}}^{9}\;{\text{M}}^{-1}\;{\text{s}}^{-1}, \end{array}$$

3.3 $$\begin{array}{r} \text{Cl}∙+\text{HOCl}\to {\text{H}}^{+}+\text{C}{\text{l}}^{-}+\text{ClO}∙,\;k_{5}=3.0\times \text{1}{\text{0}}^{9}\;{\text{M}}^{-1}\;{\text{s}}^{-1} \end{array}$$

3.4 $$\begin{array}{r} \text{and}\;\;\;\;\;\;\;\;\text{Cl}∙+\text{OC}{\text{l}}^{-}\to \text{C}{\text{l}}^{-}+\text{ClO}∙,\;k_{5}=\text{3.0}\times \text{1}{\text{0}}^{9}\;{\text{M}}^{-1}\;{\text{s}}^{-1}. \end{array}$$

At lower chlorine dosage (0.1 ∼ 1.0 mM), the enhanced generation rate of reactive radicals was greater than the increased scavenging rate by the added chlorine dosage, resulting in the increase of steady-state concentrations of reactive radicals [24]. At higher chlorine dosage (1.0 ∼ 2.0 mM), the enhanced generation rate of radicals from the increase of chlorine dosage was counterbalanced by its radical scavenging effect.

### 3.3. Effects of pH on CA degradation in UV/chlorine process

**Figure 4.**
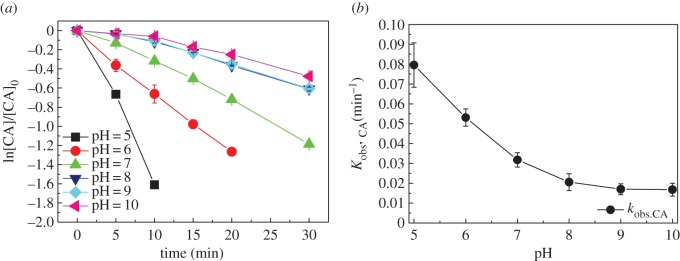
The degradation kinetics (*a*) and *k*_obs,CA_ (*b*) of CA at different pH in UV/chlorine process. Experimental conditions: *I*_0_ = 0.16 mW cm^−2^, [chlorine]_0_ = 0.8 mM, [CA]_0_ = 10 µM, *T* = 20°C.

Figure 4*a* shows the degradation kinetics of CA during UV/chlorine process at pH = 5–10, and the rate constant *k*_obs,CA_ was obtained, as shown in figure 4*b*. The *k*_obs,CA_ decreased from 0.079 min^−1^ to 0.016 min^−1^(table 1) when pH increased from 5.0 to 10.0. Free chlorine in the form of HOCl or OCl^−^ is susceptible to solution pH, which would impact the efficiency of photon absorption in UV/chlorine process [18]. The *p*K_a_ of HOCl is 7.5, thus HOCl dominates in the speciation of free chlorine at pH < 7.5 and OCl^−^ dominates at pH > 7.5. HOCl has a higher quantum yields at 254 nm than OCl^−^ (1.45 versus 0.97) [25]. On the other hand, OCl^−^ scavenges HO• with much higher rate constant (8.8 × 10^9^ M^−1^ s^−1^) than HOCl (8.46 × 10^4^ M^−1^ s^−1^) [24]. In other words, HOCl is a mild free radical scavenger contrast to OCl^−^. The effects of pH were similar to that on the degradation of trichloroethylene during UV/chlorine process as previously reported [26].

### 3.4. Contributions of HO•, RCS and UV photolysis to CA degradation in UV/chlorine process

UV photolysis and reactive radicals such as HO• and RCS may contribute to the CA degradation in UV/chlorine process. Thus, the degradation of CA in UV/chlorine process can be described as follows:

3.5 $$\frac{-\text{d[ CA] }}{\text{dt}}\text{ = }k_{\mathrm{HO}∙}[\text{CA}]\text{ + }k_{\mathrm{RCS}}[\text{CA}]\text{ + }k_{\mathrm{chlorine}}[\text{CA}]\text{ + }k_{\mathrm{UV}}[\text{CA}],$$

where *k*_HO•_, *k*_RCS_, *k*_chlorine_ and *k*_UV_ represent the first-order rate constants of CA degradation by HO• oxidation, RCS oxidation, dark chlorination and direct photolysis, respectively. Thus *k*_obs_,_CA_ degradation could be expressed as reaction (3.6), where *k*_chlorine_ is negligible (as obtained in figure 2).

3.6 $$k_{\mathrm{obs},\mathrm{CA}}=k_{\mathrm{HO}∙}+k_{\mathrm{RCS}}+k_{\mathrm{UV}}.$$

The *k*_HO•_ could be obtained by reaction (3.7), where *k′*_HO•,CA_ is the second-order rate constant of CA reacting with HO• (*k′*_HO•,CA_ = 6.98 × 10^9^ M^−1^ s^−1^), and [HO•]_SS_ is the steady-state concentration of HO•. The [HO•]_SS_ could be obtained by additional experiments using NB as a HO• probe. NB reacts with HO• at high rate constant of *k′*_HO,NB_ = 3.9 × 10^9^ M^−1^ s^−1^ and has a very low reactivity with other oxidants in the UV/chlorine system such as UV direct photolysis, chlorine and RCS [18,26]. The [HO•]_SS_ could be calculated as reaction (3.8), where *k*_obs,NB_ is the obtained first-order rate constant of NB degradation in UV/chlorine process.

3.7 $$k_{\mathrm{HO}∙}=k_{\mathrm{HO}∙,\mathrm{CA}}^{\prime }{\left[\text{HO}∙\right]}_{\mathrm{SS}}$$

and

3.8 $$k_{\mathrm{obs},\mathrm{NB}}=k_{\mathrm{HO}∙,\mathrm{NB}}^{\prime }{\left[\text{HO}∙\right]}_{\mathrm{SS}}.$$

The *k*_UV_ was obtained experimentally by UV direct photolysis of CA (stated in §3.1), then *k*_RCS_ can be calculated through *k*_obs.CA_ subtracting *k*_HO•_ and *k*_UV_. The relative contribution of each function (e.g. HO•, RCS and UV photolysis) can be obtained as *k*_HO•_/*k*_obs,CA_, *k*_RCS_/*k*_obs,CA_ and *k*_UV_/*k*_obs,CA_, respectively.

**Figure 5.**
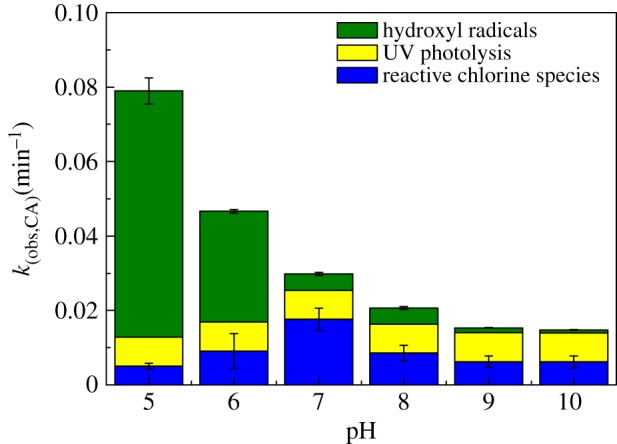
The first-order rate constants of CA degradation by HO•, RCS and UV photolysis in UV/chlorine process at different pH. Experimental conditions: *I*_0_ = 0.16 mW cm^−2^, [CA] = 10 µM, [NB] = 10 µM, [chlorine]_0_ = 0.8 mM, *T* = 20°C.

Figure 5 shows the obtained first-order rate constants of CA degradation by HO•(*k*_HO•_), RCS(*k*_RCS_) and UV photolysis (*k*_UV_) in UV/chlorine process at different pH. With increasing pH from 5 to 10, the *k*_HO•_ decreased from 0.0661 to 0.0008 min^−1^ (table 1) and the *k*_UV_ remained at 0.0078 min^−1^. The *k*_RCS_ increased from 0.0050 to 0.0176 min^−1^. This result indicates that at pH = 5, the relative contributions of HO•, RCS and UV direct photolysis to CA degradation were 84%, 6% and 10%, respectively. At neutral pH (pH = 7), the relative contribution of RCS increased to 59%, while the contribution of HO• decreased to 26%. At pH = 10, the contribution of HO• decreased to below 2%, and the contributions of UV direct photolysis and RCS were 48% and 50%, respectively. In other words, the HO• dominated CA degradation in acidic conditions, while the RCS and UV direct photolysis dominated in neutral and basic solution during UV/chlorine process.

### 3.5. Effects of water matrix on CA degradation in UV/chlorine process

**Figure 6.**
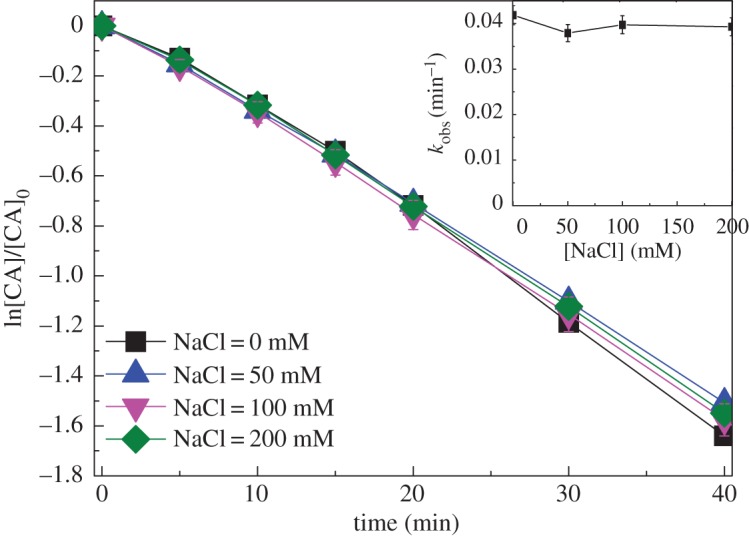
Effect of chloride on CA degradation in UV/chlorine process. Experimental conditions: *I*_0_ = 0.16 mW cm^−2^, [chlorine]_0_ = 0.8 mM, [CA]_0_ = 10 µM, pH = 7.0, *T* = 20°C.

The water matrix in surface water and wastewater, such as anions Cl^−^ and $\text{HC}{{\text{O}}_{3}}^{-}$, and HA, might influence the organic compound degradation in UV-based advanced oxidation processes [15,25,26]. The effects of Cl^−^ on CA degradation in UV/chlorine process were examined. It was found that Cl^−^ (0 ∼ 200 mM) had negligible effects on CA degradation, as shown in figure 6. This could mainly be ascribed to the following two reasons. Firstly, Cl^−^ reacts with HO• rapidly to generate HOCl^•−^ (reaction (3.9)). The HOCl^•−^ can dissociate back into HO• via the reversible reaction (reaction (3.9)), and can also react with H^+^ to form Cl• (reaction (3.10)) [23]. Under the neutral condition (pH = 7, i.e. [H^+^] = 1.0 × 10^−7^ M), the rate constant of HOCl^•−^ into Cl• (i.e. 2 × 10^10^ M^−1^ s^−1^ × 1.0 × 10^−7^ M = 2.0 × 10^3^ s^−1^) is much lower than that of HOCl^•−^ back into Cl^−^ and HO• (i.e. 6.1 × 10^9^ s^−1^) [27]. This implies that the reaction of Cl^−^ and HO• was almost negligible under the neutral pH condition [28].

3.9 $$\text{HO}∙+\text{C}{\text{l}}^{-}⇌\text{HOC}{\text{l}}^{∙-}\text{, }\;k_{+}=\text{4.3}\times \text{1}{\text{0}}^{9}\;{\text{M}}^{-1}\;{\text{s}}^{-1},\;k_{-}=\text{6.1}\times \text{1}{\text{0}}^{9}\;{\text{s}}^{-\text{1}}$$

and

3.10 $$\text{HOC}{\text{l}}^{∙-}+{\text{H}}^{+}⇌\text{Cl}∙+{\text{H}}_{2}\text{O},\;k_{+}=\text{2}\times \text{1}{\text{0}}^{\text{10}}\;{\text{M}}^{-\text{1}}\;{\text{s}}^{-\text{1}}\text{, }\;k_{-}=\text{2.5}\times \text{1}{\text{0}}^{5}\;{\text{M}}^{-\text{1}}\;{\text{s}}^{-\text{1}}.$$

Secondly, Cl^−^ reacts with Cl• resulting in the formation of Cl_2_^•−^ (reaction (1.2)). It has been reported that as Cl^−^ increased from 0 to 20 mM, the steady-state concentration of Cl_2_^•−^ increased while Cl• decreased [29]. The minimal effect of Cl^−^ on CA degradation seems likely to be ascribed to the comparable rate constants of CA with Cl_2_^•−^ and Cl•. Similar effects were observed on the degradations of carbamazepine and benzoic acid in UV/chlorine process [15,29].

**Figure 7.**
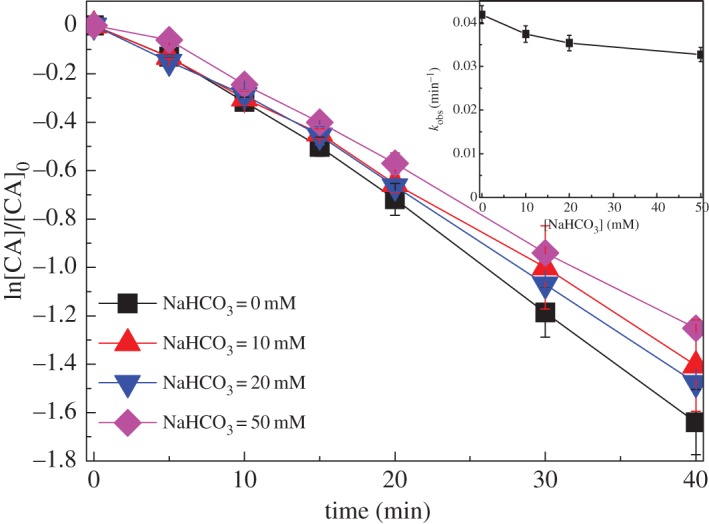
Effect of bicarbonate on CA degradation in UV/chlorine process. Experimental conditions: *I*_0_ = 0.16 mW cm^−2^, [chlorine]_0_ = 0.8 mM, [CA]_0_ = 10 µM, pH = 7.0, *T* = 20°C.

The $\text{HC}{{\text{O}}_{3}}^{-}$ (10 ∼ 50 mM) showed slight inhibition effect on CA degradation in UV/chlorine process, as shown in figure 7. The *k*_obs_ decreased from 0.041 min^−1^ to 0.032 min^−1^ (table 1) with increasing $\text{HC}{{\text{O}}_{3}}^{-}$ concentration from 0 to 50 mM. $\text{HC}{{\text{O}}_{3}}^{-}$ can react with HO• and RCS to form the carbonate radical ($\text{C}{{\text{O}}_{3}}^{∙-}$, reaction (3.11–3.13)). As a selective radical (*E*^Θ^ = 1.78 V versus NHE), $\text{C}{{\text{O}}_{3}}^{∙-}$ can oxidize organic compound through electron transfer or H-abstraction [30]. The slight inhibition effect of $\text{HC}{{\text{O}}_{3}}^{-}$ on CA degradation in UV/chlorine process might be ascribed to lower reactivity of CA with $\text{C}{{\text{O}}_{3}}^{∙-}$ than HO• and RCS. However, the second-order rate constant of $\text{C}{{\text{O}}_{3}}^{∙-}$ with CA was not obtained from the literature. Similar suppression effect of $\text{HC}{{\text{O}}_{3}}^{-}$ on CA degradation was observed in UV/H_2_O_2_ process, which was explained by lower rate constant of CA with $\text{C}{{\text{O}}_{3}}^{∙-}$ than with HO• [31].

3.11 $$\begin{array}{r} \text{HC}{{\text{O}}_{3}}^{-}+\text{HO}∙\to {\text{H}}_{2}\text{O}+\text{C}{{\text{O}}_{3}}^{∙-},\;k_{12}=\text{8.5}\times \text{1}{\text{0}}^{6}\;{\text{M}}^{-1}\;{\text{s}}^{-1}, \end{array}$$

3.12 $$\begin{array}{r} \text{HC}{{\text{O}}_{3}}^{-}+\text{Cl}∙\to {\text{H}}^{+}+\text{C}{\text{l}}^{-}+\text{C}{{\text{O}}_{3}}^{∙-},\;k_{13}=\text{2.2}\times \text{1}{\text{0}}^{8}\;{\text{M}}^{-1}\;{\text{s}}^{-1} \end{array}$$

3.13 $$\begin{array}{r} \mathrm{and}\;\;\;\;\text{HC}{{\text{O}}_{3}}^{-}+\text{C}{{\text{l}}_{2}}^{-}∙\to {\text{H}}^{+}+\text{2C}{\text{l}}^{-}+\text{C}{{\text{O}}_{3}}^{∙-},\;k_{14}=\text{8.0}\times \text{1}{\text{0}}^{7}\;{\text{M}}^{-1}\;{\text{s}}^{-1}. \end{array}$$

**Figure 8.**
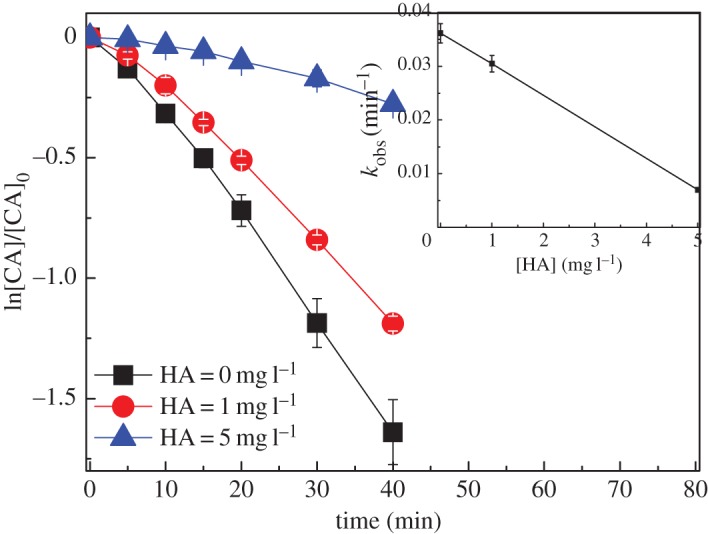
Effect of HA on CA degradation in UV/chlorine process. Experimental conditions: *I*_0_ = 0.16 mW cm^−2^, [chlorine]_0_ = 0.8 mM, [CA]_0_ = 10 µM, pH = 7.0, *T* = 20°C.

The effect of HA on CA degradation in UV/chlorine process is shown in figure 8. The CA degradation rates significantly decreased from 0.041 to 0.007 min^−1^ (table 1) with the concentration of HA increasing from 0 to 5 mg l^−1^. HA absorbs UV light at 254 nm with mole coefficient of 0.315 (l/(g cm)), thus acting as an inner filter for UV photolysis [29,32]. On the other hand, HA reacts with HO• and Cl• with second-order rate constants of 2.5 × 10^4^ (mg l^−1^)^−1^ s^−1^ and 1.3 × 10^4^ (mg l^−1^)^−1^ s^−1^ [29], respectively, thus acting as radical scavengers [33]. Li *et al.* [31] reported a similar inhibition effect of HA on CA degradation in UV/H_2_O_2_ process.

### 3.6. Degradation products and proposed degradation pathways of CA in UV/chlorine process

**Figure 9.**
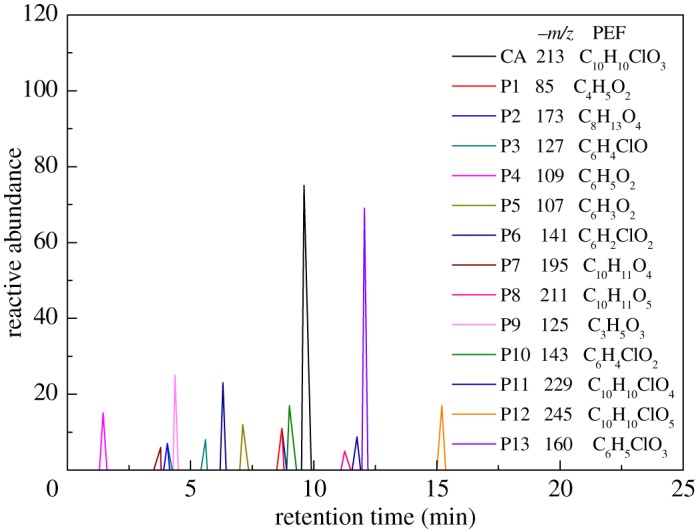
Extracted total ion chromatogram (TIC) of CA in UV/chlorine process. Experimental conditions: *I*_0_ = 0.16 mW cm^−2^, [CA]_0_ = 300 μM, [chlorine]_0_ = 1.6 mM, pH = 7.0, *T* = 20°C.

**Figure 10.**
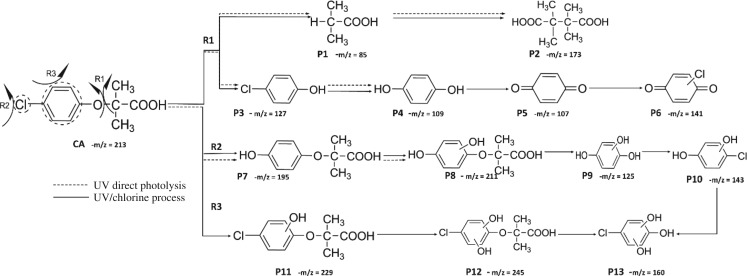
The degradation pathways of CA in UV photolysis and UV/chlorine process. Experimental conditions: *I*_0_ = 0.16 mW cm^−2^, [CA]_0_ = 300 µM, [chlorine]_0_ = 1.6 mM, pH = 7.0, *T* = 20°C.

The HPLC GE-MS/MS total ion chromatogram (TIC, negative ionization mode) for CA degradation in UV/chlorine process is shown in figure 9. The TIC gave the chromatographic retention time, accurate mass to charge ratios (−*m/z*) and proposed empirical formula (PEF) of intermediates. The structures of intermediates were proposed according to the PEF and MS/MS spectra of fragments and listed in table 2. Thirteen different intermediates of CA degradation in UV/chlorine process were identified. According to the structures of the intermediates and previously reported degradation pathways in other processes [4,34,35], three main pathways (**R1–R3**) for CA degradation in UV/chlorine process (solid lines) were proposed, as shown in figure 10.

**Table 2.** The degradation products of CA in UV photolysis and UV/chlorine process.

					detected in	
degradation products	proposed empirical formula	−*m/z*	retention time (min)	proposed structure	UV photolysis	UV/chlorine process
CA	C_10_H_10_ClO_3_	213	9.66	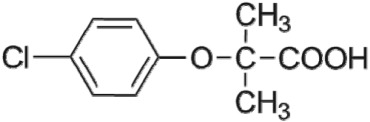	√	√
P1	C_4_H_5_O_2_	85	8.7	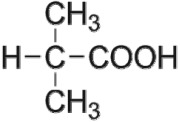	√	√
P2	C_8_H_13_O_4_	173	4.05	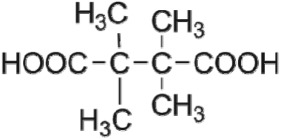	√	√
P3	C_6_H_4_ClO	127	5.6	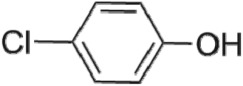	√	√
P4	C_6_H_5_O_2_	109	1.45	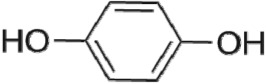	√	√
P5	C_6_H_3_O_2_	107	7.12	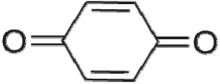		√
P6	C_6_H_2_ClO_2_	141	6.31	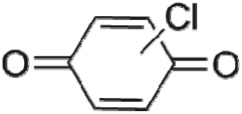		√
P7	C_10_H_11_O_4_	195	3.78	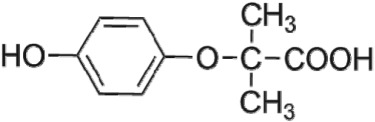	√	√
P8	C_10_H_11_O_5_	211	11.26	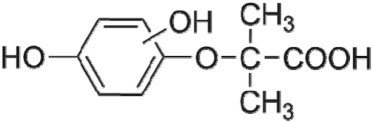	√	√
P9	C_3_H_5_O_3_	125	4.35	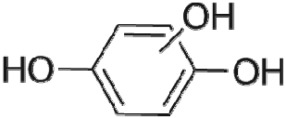		√
P10	C_6_H_4_ClO_2_	143	9.01	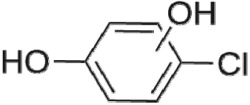		√
P11	C_10_H_10_ClO_4_	229	11.75	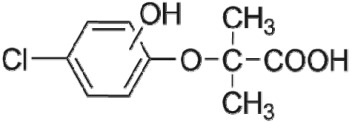		√
P12	C_10_H_10_ClO_5_	245	15.2	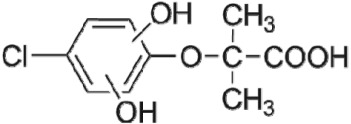		√
P13	C_6_H_5_ClO_3_	160	12.06	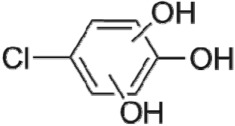		√

The first way termed as **aryloxy-carbon cleavage route (R1)**. CA (−*m/z* = 213) was broken at the site of aryloxy-carbon into P1 (methacrylate, −*m/z* = 85) and P3 (4-chlorophenolate, −*m/z* = 127), which were two identified informative fragment ions. These two fragments were also detected in CA degradation during pulse radiolysis process [35]. In addition, the polymerized compound (P2, −*m/z* = 173) was probably formed after the further oxidation of P1 via H-abstraction. P3 was further oxidized via attacking at the site of aryl-halogen yielding P4 (hydroquinone, −*m/z* = 109), which was frequently detected during aromatic compounds oxidation [4]. P4 was oxidized to benzoquinone (P5, −*m/z* = 107) via two successive one-electron transfer processes, and the main active site is *para*-C–OH bond which is suggested to be innate and high reactive [36,37]. High electron cloud density of the C=C double in benzoquinone may have resulted in further –Cl substitution of P5 yielding *ortho*-chlorinated phenols P6 (−*m/z* = 141) [38,39].

The second way termed as **aryl-halogen cleavage route (R2).** The aryl-halogen (C–Cl) of CA can be attacked by HO•, resulting in the cleavage of chloride atom, yielding a dechlorinated intermediate 2-(4-hydroxyphenoxy)-isobutyric acid (P7, −*m/z* = 195) [36,40]. P7 is a phenolic substance that is susceptible to be further oxidized by HO• to produce dihydroxyl P8 (−*m/z* = 211) [35]. The aryloxy-carbon bond of P8 is fragile and easily broken to form phloroglucinol (P9, −*m/z* = 125). The *para*-C–OH bond of P9 incurred –Cl substitution, yielding 4-chlorosercinol (P10, −*m/z* = 143) [35,41]. The *ortho*-hydroxy-substituted carbons of P10 can be further oxidized by the addition of a –OH to the aryl ring forming P13 (−*m/z* = 160) [41]. The reaction products and pathways of P1, P3–P5, P7–P10 were also reported in other processes for degradation of CA [4,34,35,42].

The third way termed as **aryl ring addition (R3).** The electrophilic radical (Cl•) prefers to attack the *para*-substituted aromatic ring via OH-addition [42,43], resulting in the formation of primary intermediate P11 (−*m/z* = 229). P11 can be further oxidized into P12 (−*m/z* = 245) via similar addition of –OH on the aryl ring [35]. The cleavage of aryloxy-carbon in P12 resulting in the formation of P13 (−*m/z* = 160) [35], which is a trihydroxyl phenol compound with chlorine substitution. P13 has an isomer of 5-chloro-1,2,4-benzenetriol, which has been reported to be the major product during the degradation of 4-chlorophenol (P3) in TiO_2_-mediated photocatalytic process [44]. P13 has the largest relative abundance as shown in figure 9, and was observed after 3 h irradiation under five-time excessive chlorine. Above all, P13 was likely to be the main final degradation product of CA in UV/chlorine process [45].

UV photolysis also contributed to the CA degradation in UV/chlorine process, as discussed in §3.1. During UV direct photolysis of CA, six intermediates (P1–P4, P7, P8) were identified, which were the primary products formed in UV/chlorine process, as shown in figure 10 (dotted lines).

## 4. Conclusion

This study investigated the kinetics and pathways of CA degradation in UV/chlorine process. Compared with UV photolysis, CA degradation was greatly enhanced in UV/chlorine process, while CA could not be degraded by dark chlorination. With the chlorine dosage increasing, the *k*_obs,CA_ increased quickly until reaching a plateau. The *k*_obs,CA_ in UV/chlorine process reduced gradually as pH increased from 5.0 to 10.0. The HO• dominated CA degradation in acidic conditions while the RCS and UV photolysis dominated in neutral and basic solution. The degradation of CA was slightly inhibited by HCO_3_^−^, and significantly inhibited by HA in UV/chlorine process. However, the presence of Cl^−^ showed no effect on CA degradation. These kinetic results could be used to predict the CA behaviour in the water treatment process, thus guiding the practical application. Furthermore, the degradation intermediates were monitored by LC/MS/MS, and the proposed degradation pathways suggested that aryloxy-carbon cleavage, aryl-halogen cleavage and aryl ring substitution were the main degradation pathways of CA in UV/chlorine process. These degradation pathways suggested that the primary intermediates formed in UV photolysis process do not have any chlorinated disinfection by-products (DBPs), but they could be further oxidized into DBPs in UV/chlorine process. Therefore, it may increase the bio-risk and toxicity of CA degradation products after UV/chlorine process, which needs to be evaluated in further studies.

## Data accessibility

All relevant data are within the paper.
